# HIV and hepatitis C virus test uptake at methadone clinics in Southern China: opportunities for expanding detection of bloodborne infections

**DOI:** 10.1186/1471-2458-13-899

**Published:** 2013-09-30

**Authors:** Ying-Hua Xia, Wen Chen, Joseph D Tucker, Charles Wang, Li Ling

**Affiliations:** 1 Faculty of Medical Statistics and Epidemiology, School of Public Health, Sun Yat-sen University, No. 74, Zhongshan Road II, Yuexiu District, Guangzhou 510080, P.R. China; 2 Sun Yat-sen Center for Migrant Health Policy, Sun Yat-sen University, No. 74, Zhongshan Road II, Yuexiu District, Guangzhou 510080, P.R. China; 3 UNC Project-China, Guangdong STD Control Center, Guangzhou 510095, P.R. China; 4 Division of Gastroenterology, Rhode Island Hospital / Alpert Medical School of Brown University, 593 Eddy Street, APC 414, Providence, RI 02903, USA

**Keywords:** Bloodborne infections, Testing uptake, Methadone maintenance treatment clinic

## Abstract

**Background:**

HIV and hepatitis C (HCV) co-infection is highly common among Chinese injection drug users but it is difficult to reach IDUs at traditional VCT (Voluntary HIV counseling treatment) clinics. A new national model integrating HIV/HCV testing with methadone maintenance treatment was started in 2006. The purpose of this study was to investigate HIV and HCV test uptake and associated factors at methadone clinics in Guangdong Province, China.

**Methods:**

A cross-sectional design using routine surveillance data and laboratory testing confirmation was applied to determine rates of HIV and HCV test uptake. Multi-level modeling was used to examine individual-level and clinic-level correlates of increased test uptake.

**Results:**

45 out of 49 methadone clinics in Guangdong Province agreed to participate in the study. Among all 13,270 individuals, 10,046 (75.7%) had HIV test uptake and 10,404 (78.4%) had HCV uptake. At the individual level, methadone clients 30 years or older were more likely to have HIV and HCV test uptake (p <0.001 for both). At the clinic level, methadone clinics with greater health care personnel were more likely to have HIV (p =0.01) and HCV (p = 0.044) test uptake. HIV test uptake significantly correlated with HCV test uptake (correlation coefficient=0.64).

**Conclusion:**

Methadone clinics provide an opportunity for routine integrated HIV and HCV screening among drug users in China. Increased test uptake in young drug users and increased health care personnel at clinics may further improve screening.

## Background

Early HIV testing and treatment are critical to the success of comprehensive control programs, especially among most-at-risk populations [1,2]. Although HIV testing is offered in a variety of health care settings across the world, poor test uptake among drug users continues to be a common problem worldwide [3,4]. Currently less than half of HIV-infected individuals in China know their serostatus among the estimated 780,000 HIV-infected individuals [5]. Modeling research has suggested that increasing HIV testing fourfold in China could avert over 42,000 HIV infection and 11,000 deaths [6]. Scaling up HIV testing has been challenging in China. Traditional voluntary HIV counseling treatment (VCT) testing has failed to reach many drug users in China. Less than 20% of drug users in China received HIV testing at VCT sites [7,8].

HIV and hepatitis C (HCV) co-infection is common among Chinese injection drug users (IDUs) [9]. In one study from southern China, 17.6% of IDUs had HCV infection and in 95.1% of HCV-infected individuals also had HIV infection [10]. Since HIV/HCV co-infection increases mortality, cirrhosis, and hepatocellular carcinoma, scaling up integrated HIV and HCV testing is an important public health priority [11].

To address these challenges, in 2006 China started a model of HIV/HCV screening among drug users at methadone maintenance treatment clinics (methadone clinics) [12,13]. Free voluntary routine screening for HIV and HCV infection were integrated into methadone clinics and provided to all clients, although individuals could still decline testing. Currently 700 methadone clinics across China distribute methadone to 300,000 drug users and coverage continues to expand [14,15].

However, the extent to which this model successfully implements HIV and HCV testing in China is currently unknown. The purpose of this study was to examine HIV and HCV test uptake at methadone clinics in Guangdong Province, China and to identify individual and clinic level factors associated with increased uptake.

## Methods

### Study site

Guangdong Province is located in southern China and has the largest migrant population of any province [16]. The province has a system of 49 methadone clinics. Methadone clients are self-referred or assigned treatment as part of a post-incarceration rehabilitation plan [13]. The system is administered by the National Centre for AIDS/STD Control and Prevention within the Chinese Centers for Disease Control and Prevention (Chinese CDC). The system has associated clinics that distribute methadone to recovering drug users. Guangdong’s 49 methadone clinics are distributed in 18 of the 23 cities of the province.

### Administrative management system

A national web-based national HIV/AIDS information system was established by the Chinese CDC and includes methadone maintenance treatment as one of its eight core subsystems [17].

The methadone clinic administrative database has detailed records of all individuals, including socio-demographic characteristics, drug use behaviors, and HIV and HCV test uptake. Test uptake was defined as laboratory confirmed completion of an HIV Western Blot or HCV ELISA test. All surveys were completed by local methadone clinic staff and entered into the national web-based administrative management system. Survey items were developed by the National Working Group for Community-based Maintenance Treatment for Opiate Users [13].

### Data source

We conducted a performance evaluation of the methadone maintenance program in Guangdong Province. Data for all 49 methadone clinics opened before October 31, 2008 were collected during November and December 2008. Methadone clinic staff downloaded data and deleted all identifying information.

The director of each methadone clinic completed a detailed questionnaire about their clinic including economic region, administrative clinic level, affiliated institution, clinic healthcare personnel (the total number of nurses and physicians employed), number of patients, integration of clinic with voluntary HIV counseling services, clinic finances (for profit or not), and average distance from client residence to clinic (within five kilometers or further).

### Data analysis

A total of 49 clinics were examined. Four clinics open less than one month prior to the study were not included in the analysis. 172 (1.3%) individuals were excluded from the analysis because they had three or more individual-level variables missing in their data set. Another 296 (2.2%) individuals were excluded because they initiated methadone maintenance treatment in another province before transferring their care to Guangdong.

The main study outcomes were HIV test uptake and HCV test uptake. Group comparisons for categorical data were examined with Chi-square tests at the individual level. HIV and HCV test uptake were examined using nonparametric Kruskal-Wallis tests at the clinic level. The correlation between HIV test uptake and HCV test uptake was examined using a Pearson correlation coefficient.

Given the hierarchical nature of the data, there was potential clustering in outcomes and interactions between individual and clinic level factors of HIV and HCV test outcomes. Multilevel analysis with random intercepts was used to assess the effect of factors at the individual and clinical level on HIV and HCV test uptake. To examine clustering at the clinic level, a random intercept two-level null model with no explanatory variables was first tested. Results were consistent with clustering at the clinic level, supporting the use of a multilevel model.

The multilevel analysis used a two-level binomial logit model that incorporated six individual level (level 1) variables and eight clinic level variables (level 2). Individual level socio-demographic characteristics included the following: sex, age (<30 years old, 30–39 years old, 40–49 years old, >50 years old), marital status (married, unmarried), education completed (none/primary school, middle school, high school and above), and employment status (employed, unemployed). Behavioral data in the system included major drug type, injecting behaviors (injecting, non-injecting), drug use method during the past half year (injecting, smoking/snorting, mixing injecting and smoking/snorting), needle sharing experience (sharing, non-sharing) and detoxification experience (ever had detoxification, never). Only drug injecting experience was included in the analysis. Major drug type and detoxification experience were not included because more than 90% of individuals were heroin addicts and almost all of them had detoxification experience. Drug use during the past half year and needle sharing experience were excluded since they were highly correlated with injecting drug experience. All clinic level variables mentioned above were included. Clinic healthcare personnel (physicians and nurses) and total number of individuals at each clinic were fitted as continuous variables. The form of the binomial logit 2-level model and interpretation are described elsewhere [18]. Stepwise selection was used to choose the variables for the final model, first using individual level variables and then using clinic level variables. The final model was designed by first including all variables in the model that were significant and then examining the best fit based on –2log-likelihood values [19]. Models were created using discrete variables as dummy or grouped continuous variables and -2log-likelihood values were compared.

Analysis used SAS 9.1 (SAS Institute Inc, Cary, NC, USA) except for the multilevel analysis which used MLwiN 2.02 (http://www.cmm.bristol.ac.uk/MLwiN/). Reported p-values were two-sided and a p-value below 0.05 was judged significant.

### Ethics statement

This study was approved by the Guangdong Provincial Health Bureau and the Ethics Review Committee at Sun Yat-sen University. Written informed consent was provided by clinic directors and all patients. The patient consent included permission to upload information into the national web-based administrative database.

## Results

### Socio-demographic characteristics of participants and characteristics of clinics

A total of 13,270 individuals were included in this analysis. Most individuals were male (93.9%) and heroin users (99.6%). 82.6% were between 20 years old and 40 years old. Most individuals had completed nine years or less of school and were unemployed at the time of the survey (Table 1).Twenty-five out of the 45 clinics were located in the prosperous Pearl River Delta region of the province. 82.2% of clinics were at the city level. The median total number of methadone clients seen in each clinic was 280 (range 21–1029). The median number of healthcare personnel at each clinic was 5 (range 2–10) (Table 2).

**Table 1:** Individual-level characteristics associated with HIV and HCV test uptake at methadone clinics (N=13270)

**Variables**	**No. of patients (%)**	**HIV uptake**		**HCV uptake**	
		**No. (%)**	**p**	**No. (%)**	**p**
Overall	13270(100)	10046(75.7)		10404(78.4)	
Sex					
Male	12463(93.9)	9400(75.4)	0.003	9753(78.3)	0.106
Female	807(6.1)	646(80.1)		651(80.7)	
Age					
<20 years old	36(0.3)	25(69.4)	<0.001	25(69.4)	<0.001
20-29 years old	3242(24.4)	2346(72.4)		2411(74.4)	
30-39 years old	7725(58.2)	5907(76.5)		6148(79.6)	
40-49 years old	2080(15.7)	1621(77.9)		1664(80.0)	
50-59 years old	177(1.3)	140(79.1)		149(84.2)	
>59 years old	10(0.1)	7(70.0)		7(70.0)	
Marital status					
Married	6446(48.6)	4911(76.2)	0.208	5079(78.8)	0.288
Unmarried	6824(51.4)	5135(75.3)		5325(78.0)	
Education					
None/primary school	2760(20.8)	1695(77.6)	0.049	2158(78.2)	0.661
Middle school	8325(62.7)	6292(75.6)		6517(78.3)	
High school and above	2185(16.5)	2059(74.6)		1729(79.1)	
Employment status					
Employed	4926(37.1)	3752(76.2)	0.340	3650(74.1)	<0.001
Unemployed	8344(62.9)	6294(75.4)		6754(80.9)	
Ever injected drugs					
Yes	11011(83.0)	8348(75.8)	0.512	8663(78.7)	0.091
No	2259(17.0)	1698(75.2)		1741(77.1)	

Note: uptake is defined as completion of HIV or HCV test at an associated laboratory.

**Table 2:** Clinic-level characteristics associated with HIV and HCV test uptake at methadone clinics (N=45)

**Variables**	**No. of clinics (%)**	**HIV test uptake (%)**			**HCV test uptake (%)**		
		**Median**	**P**_**25**_**-P**_**75**_	***P***	**Median**	**P**_**25**_**-P**_**75**_	***P***
Overall	45(100)	86.4	71.8-98.6		94.9	83.1-97.8	
Economic region							
Pearl River Delta*	25(55.6)	87.1	79.5-98.7	0.209	94.9	85.0-97.9	0.385
Non-Pearl River Delta	20(44.4)	84.8	57.9-97.0		90.4	71.0-97.5	
Administrative clinic level							
Town/village level	8(17.8)	83.1	79.2-97.1		96.8	57.3-97.8	
City level	37(82.2)	86.8	63.9-98.7	0.894	92.4	83.1-97.8	0.859
Affiliated institution**							
Center of Disease Control (CDC)	13(28.9)	86.8	72.9-97.7	0.679	96.1	84.5-96.9	0.940
Non-CDC	32(71.1)	85.2	63.3-98.7		93.6	82.4-97.9	
Clinic healthcare personnel							
2-4 staff	13(28.9)	63.9	54.1-89.7	0.079	83.1	71.0-95.0	0.154
5-7 staff	27(60.0)	86.2	72.9-98.6		96.4	84.7-98.3	
8-10 staff	5(11.1)	97.7	89.8-98.9		96.6	90.9-99.6	
Total number of patients							
<200 patients	17(37.8)	89.7	72.9-97.7	0.462	96.6	86.7-97.9	0.345
200-399 patients	17(37.8)	86.4	63.9-98.8		92.4	84.5-97.5	
>=400 patients	11(24.4)	82.8	35.0-98.0		84.7	31.8-98.3	
Provided voluntary HIV counseling or not							
Yes	29(64.4)	86.8	78.9-98.7	0.484	95.0	84.5-97.9	0.652
No	16(35.6)	84.0	57.9-96.8		92.9	81.1-97.4	
Average distance from clients’ residence to clinic							
≤5 km	20(44.4)	86.8	62.7-99.0	0.991	88.4	75.0-97.7	0.615
>5 km	25(55.6)	86.2	72.9-97.7		96.1	83.3-97.8	
Clinic made profit or not							
Yes	19(42.2)	86.2	63.4-98.8	0.973	96.4	85.2-97.8	0.491
No	26(57.8)	86.6	72.1-97.7		88.8	81.8-97.9	

*Pearl River Delta: A dense network of economically developed cities in South China.

**Affiliated institution: A methadone clinic is not an independently operated institution. Clinics are affiliated to institutions such as a local Center of Disease Control, public comprehensive hospital or specialist hospital. The affiliated institution manages the clinic, becomes responsible for administrative issues, and provides human resources and equipment to the clinic.

### Individual-level characteristics associated with HIV and HCV test uptake

Among all 13,270 individuals, 10,046 (75.7%) had HIV test uptake and 10,404 (78.4%) had HCV uptake. Among IDUs and non-IDUs, the HIV prevalence was 7.0% and 0.8%, respectively. Among IDUs and non-IDUs, the HCV prevalence was 75.5% and 32.6%, respectively. Several individual factors were significantly associated with HIV test uptake and HCV test uptake using univariate analysis (Table 1). Women had a HIV uptake rate of 80.1% compared to men who had an HIV uptake rate of 75.4% (P=0.003). HIV uptake and HCV uptake were increased among older individuals (P<0.001). Individuals younger than twenty years old had the lowest HIV and HCV uptake (both were 69.4%) and individuals between 50 to 59 years old had the highest HIV and HCV uptake (79.1% and 84.2%, respectively). Individuals who were unemployed had higher HCV uptake (80.9%) than individuals who were employed (74.1%) (P<0.001).

### Clinic level characteristics associated with HIV and HCV test uptake

At the clinic level, the median HIV test uptake among 45 clinics was 86.4% (P_25_-P_75_: 71.8%-98.6%) and the median HCV test uptake was 94.9% (83.1%-97.8%). No factors were significantly associated with clinic test uptake based on the univariate analysis (Table 2).

### HIV test uptake – individual and clinic level associations

The final two-level logit model showed age, sex, drug injecting experience and clinic healthcare personnel were associated with HIV test uptake. Younger individuals were less likely to have HIV test uptake than older individuals. Non-injecting drug users were more likely to have HIV test uptake than IDUs (OR=1.16, 95% CI=1.01-1.33). Women had higher HIV uptake than men (OR=1.36, 95%=1.08-1.70). Clinics with greater healthcare personnel had higher HIV uptake (OR=1.55, 95% CI=1.09-2.20) (Table 3).

**Table 3:** Factors associated with HIV test uptake using multilevel model

**Parameters**	**Estimate(s.e.)**	**Odds ratio (95% confidence interval)**	**P**
Null model			
Random			
Level 2			
$\sigma_{\mu_{O}}^{2}$	4.227(0.947)		<0.001
Model with explanatory variables			
Random			
Level 2			
$\sigma_{\mu_{O}}^{2}$	3.720(0.854)		<0.001
Fixed			
Age			
<30 years old	-	1	
30-39 years old	0.229(0.062)	1.26(1.11,1.42)	<0.001
40-49 years old	0.490(0.087)	1.63(1.38,1.94)	<0.001
>50 years old	0.401(0.231)	1.49(0.95,2.35)	0.082
Sex			
Male	-	1	0.007
Female	0.305(0.114)	1.36(1.08, 1.70)	
Ever injected drugs			
Yes	-	1	0.037
No	0.147(0.070)	1.16(1.01, 1.33)	
Clinic healthcare personnel*	0.439(0.178)	1.55(1.09, 2.20)	0.013

*: Healthcare personnel are defined as the total number of physicians and nurses at the clinic.

### HCV test uptake – individual and clinic level associations

As shown in Table 4, the final model identifying correlates of HCV test uptake included age, employment status and clinic healthcare personnel. Similar to HIV test uptake data, older individuals were more likely to have HCV test uptake than younger individuals (the OR of 30–39 and 40–49 versus under 30 years are 1.29 (95% CI=1.13-1.49) and 1.54 (95% CI=1.27-1.88)). More healthcare personnel at the methadone clinic were associated with higher HCV test uptake (OR=1.46, 95% CI= 1.01-2.11). Unemployed individuals were more likely to have HCV test uptake than employed individuals (OR=1.18, 95% CI=1.03-1.34).

**Table 4:** Factors associated with HCV test uptake using multilevel model

**Parameters**	**Estimate(s.e.)**	**Odds ratio (95% confidence interval)**	**P**
Null model			
Random			
Level 2			
$\sigma_{\mu_{O}}^{2}$	4.427(0.991)		<0.001
Model with explanatory variables			
Random			
Level 2			
$\sigma_{\mu_{O}}^{2}$	4.103(0.942)		<0.001
Fixed			
Age			
<30 years old	-	1	
30-39 years old	0.258(0.071)	1.29(1.13,1.49)	<0.001
40-49 years old	0.433(0.100)	1.54(1.27,1.88)	<0.001
>50 years old	0.725(0.302)	2.06(1.14,3.73)	0.016
Employment status			
Employed	-	1	0.014
Unemployed	0.161(0.066)	1.17(1.03, 1.34)	
Clinic healthcare personnel*	0.379(0.188)	1.46(1.01, 2.11)	0.044

*: Healthcare personnel are defined as the total number of physicians and nurses at the clinic.

### Correlation between HIV and HCV uptake

There was a significant correlation between HIV test uptake and HCV test uptake. The correlation coefficient between HIV test uptake and HCV test uptake was 0.64 (P<0.001). Among the 11,254 individuals who received either test, 1208 (10.7%) received only HIV testing or HCV testing, but not both. Women (OR 1.57(95% CI=1.14-2.15)), individuals 40 to 49 years old (OR 1.53 (95% CI=1.20-1.94)), and non-IDUs (OR 1.42 (95% CI=1.16- 1.74)) were more likely to have received both HIV and HCV testing (Table 5).

**Table 5:** Factors associated with testing for only HIV or HCV using multilevel model (N=11254)

**Parameters**	**Rate of testing (%)**	**Estimate(s.e.)**	**Odds ratio (95% confidence interval)**	**P**
Null model				
Random				
Level 2				
$\sigma_{\mu_{O}}^{2}$		2.913(0.629)		<0.001
Model with explanatory variables				
Random				
Level 2				
$\sigma_{\mu_{O}}^{2}$		2.866(0.619)		<0.001
Fixed				
Age				
<30 years old	10.7	-	1	
30-39 years old	10.9	0.166(0.089)	1.18(0.99, 1.41)	0.0617
40-49 years old	10.0	0.424(0.122)	1.53(1.20, 1.94)	<0.001
>50 years old	12.0	0.027(0.283)	1.03(0.59, 1.79)	0.924
Sex				
Male	10.9	-	1	
Female	8.2	0.448(0.161)	1.57(1.14, 2.15)	0.005
Ever injected drugs				
Yes	11.0	-	1	<0.001
No	9.5	0.349(0.104)	1.42(1.16, 1.74)	

## Discussion

This study reveals an opportunity to expand detection of bloodborne infections at methadone clinics in China. To our knowledge, this study is the first to examine integrated HIV and HCV test uptake in Chinese methadone clinics. China’s model of routine screening at methadone clinics expanded access to HIV and HCV test among drug users, but our study found more than 20% of individuals seen at methadone clinics were still not screened for HIV or HCV. Older clients and more healthcare personnel at clinics were both associated with a higher test uptake. Our large sample size and incorporation of clinic level factors expands on previous research in this field [8,20,21].

We found a high rate of HIV and HCV test uptake at methadone clinics. HIV test uptake rates in our study were similar to HIV test uptake rates reported in VCT clinics (72.7%) and STI clinics (81.2%) [18,22]. In China, methadone clinics are well positioned to scale up testing because many new HIV and HCV infections are acquired through drug use [7]. We found an HCV prevalence (75.5%) higher than reported in a national systemic review (66.97%) [9]. Free HCV testing at methadone clinics eliminates a potential barrier to screening associated with clinic-based testing. One study showed IDUs preferred methadone clinics more than general medicine clinics or specialized clinics as locations for HIV and HCV testing [23]. Our research suggests that routine HIV/HCV screening at methadone clinics can provide a successful model for scaling up HIV/HCV prevention and control programs.

We found younger individuals at methadone clinics had poor HIV and HCV test uptake. While other Chinese studies have not observed the same trend [18,24], our findings are consistent with the literature outside of China. Suboptimal HIV test uptake among youth has also been noted in the United States, Thailand, and Canada [25-27]. Poor awareness of infection, fear of knowing the results, and fear of stigma may be exacerbated among drug using youth [28-30] and were correlated with not receiving HIV testing among young people [31-33]. Since youth drug abuse is becoming more common in China [12] and HIV and HCV infection among young drug users are increasing [10,34], developing tailored counseling for young individuals at methadone clinics may improve HIV and HCV test uptake.

Our findings suggest that lack of healthcare personnel may constrain HIV and HCV testing at methadone clinics. After adjusting for individual and clinic level factors, clinics with a higher number of healthcare personnel (physicians and nurses) were more likely to have HIV and HCV test uptake. Fewer healthcare personnel contribute to relatively heavier workloads and may lead to inadequate counseling during routine testing [35]. National methadone clinic guidelines require each clinic have two attending physicians and two nurses with training in mental health, HIV counseling, and methadone maintenance [13]. Our research found only 23.3% and 34.9% of methadone clinics in Guangdong province met these physician and nurse staffing requirements, respectively [36]. Other research suggests limited staffing is a problem throughout China [35] and to a lesser extent, the U.S [37]. Often two or three part-time clinicians are managing care for more than 100 patients, including writing prescriptions, providing counseling and health education, tracking patients lost to follow-up, and preparing reports [38]. Limited staffing may contribute to low quality counseling service, a known barrier to HIV testing [15,24]. Increasing the number of methadone clinic nurses may help expand routine HIV and HCV testing.

This study has several limitations. First, our use of routine monitoring data did not identify reasons for non-uptake. Although several correlates were associated with non-uptake, the broader social context of this missed public health opportunity can only be inferred. Second, our data were obtained from a single province in South China. However, Guangdong Province is diverse and draws individuals from a broad number of provinces and regions throughout China. Third, some individuals have HIV/HCV testing in other rehabilitation settings before methadone treatment, leading to individuals declining repeat testing. Research examining methadone clients perspectives is necessary to comprehensively interpret our results. Fourth, the data was collected at the end of 2008, but national policies have not changed since then. Finally, we have no data on the extent of test counseling at each clinic. Measuring this process outcome is important for ensuring that testing can be expanded.

## Conclusion

Our findings emphasize the need for future research on test uptake and delivery of services in China’s methadone clinics. Greater attention to young individuals and methadone clinic personnel are warranted. Increasing healthcare personnel at methadone clinics may increase test uptake. The use of multilevel modeling allowed examination of clinic level attributes of test uptake.

## Competing interests

The authors declared that they have no competing interests.

## Authors’ contributions

YX contributed to paper writing and data analysis. WC contributed to data collecting and data analysis. JDT contributed to paper writing and analysis. CW contributed to paper writing. The corresponding author, LL, contributed to funding application and supervision on project conducting. All authors read and approved the final manuscript.

## Pre-publication history

The pre-publication history for this paper can be accessed here:

http://www.biomedcentral.com/1471-2458/13/899/prepub
